# Supplementary material for: Framework for quality assessment of whole genome cancer sequences
Source: Nat Commun. 2020 Oct 7;11:5040. doi: 10.1038/s41467-020-18688-y (PMC7541455; doi:10.1038/s41467-020-18688-y)
Supplement: Supplementary file 1 — Supplementary Information [file 41467_2020_18688_MOESM1_ESM.pdf]

Framework for quality assessment of whole genome, cancer sequences

Justin P. Whalley[1,2], Ivo Buchhalter[3,4,5,6], Esther Rheinbay[7,8], Keiran M. Raine[9], Miranda D. Stobbe[1], Kortine Kleinheinz[3], Johannes Werner[3,10], Sergi Beltran[1], Marta Gut[1], Daniel Hübschmann[3,4,11,12,13], Barbara Hutter[6], Dimitri Livitz[7], Marc D. Perry[14], Mara Rosenberg[7,8], Gordon Saksena[7], Jean-Rémi Trotta[1], Roland Eils[15,16], Daniela S. Gerhard[17], Peter J. Campbell[9], Matthias Schlesner[3,18] & Ivo G. Gut*[1,19]

[1]*CNAG-CRG, Centre for Genomic Regulation (CRG), Barcelona Institute of Science and Technology (BIST), Baldiri i Reixac 4, 08028 Barcelona, Spain*

[2]*Present address: Wellcome Centre for Human Genetics, University of Oxford, Roosevelt Drive, Oxford, United Kingdom*

[3]*Division of Theoretical Bioinformatics (B080), German Cancer Research Centre (DKFZ), Heidelberg, Germany*

[4]*Department for Bioinformatics and Functional Genomics, Institute for Pharmacy and Molecular Biotechnology (IPMB) and BioQuant, Heidelberg University, Heidelberg, Germany*

[5]*Omics IT and Data Management Core Facility (W610), German Cancer Research Center (DKFZ), Heidelberg, Germany*

[6]*Division of Applied Bioinformatics (G200), German Cancer Research Centre (DKFZ), Heidelberg, Germany*

[7]*Broad Institute of Harvard and MIT, Cambridge, MA, USA*

[8]*Massachusetts General Hospital Cancer Center and Department of Pathology, Boston, MA, USA*

[9]*Wellcome Sanger Institute, Hinxton, UK*

[10]*Present address: Department of Biological Oceanography, Leibniz Institute of Baltic Sea Research, Seestraße 15, Rostock, Germany*

[11]*Department of Pediatric Immunology, Hematology and Oncology, University Hospital Heidelberg, Heidelberg, Germany*

[12]*Present address: Computational Oncology, Molecular Diagnostics Program, National Center for Tumor diseases (NCT) Heidelberg and German Cancer Research Center (DKFZ), Heidelberg, Germany*

[13]*Present address: Heidelberg Insititute for Stem cell Technology and Experimental Medicine (HI-STEM), Heidelberg, Germany*

[14]*Department of Radiation Oncology, University of California, San Francisco, USA*

[15]*Center for Digital Health, Berlin Institute of Health (BIH) and Charité - Universitätsmedizin Berlin, corporate member of Freie Universität Berlin, Humboldt-Universität zu Berlin, Berlin, Germany*

[16]*Health Data Science Unit, Heidelberg University Hospital and BioQuant, Im Neuenheimer Feld 267, 69120 Heidelberg, Germany*

[17]*Office of Cancer Genomics, National Cancer Institute, US National Institutes of Health, Bethesda, MD, USA*

[18]*Bioinformatics and Omics Data Analytics (B240), German Cancer Research Centre (DKFZ), Heidelberg, Germany*

[19]*Universitat Pompeu Fabra (UPF), Barcelona, Spain*

**Corresponding author: ivo.gut@cnag.crg.eu*

Supplementary Information

List of Supplementary Figures

1 Mean insert size for normal and tumour samples. 4

2 Histogram of the mean coverage for normal and tumour samples 5

3 Histogram of the median over mean coverage ratios for normal and tumour samples 6

4 Histogram of the FWHM measures for normal and tumour samples 7

5 Histogram of the somatic mutation calling coverage values for normal-tumour sample pairs 8

6 Histogram of the percentage of paired reads mapping to different chromosomes for normal and tumour samples 9

7 Histogram of the ratios of difference in edits between paired reads for normal and tumour samples 10

8 Histogram of the star ratings for the PCAWG genomes, as grouped by organ system 11

9 Increase in quality of the CLLE-ES samples sequenced in 2012/13 compared to 2010/11. 12

10 Quality of the samples sequenced at the Broad Institute over time. 13

List of Supplementary Tables

1 Table showing that somatic mutation callers were in greater agreement for cancer genome sequences with 4 stars or more. 14

2 Results for the linear regression model showing the relationship between calling somatic single base mutations (SSM) and the QC measures. 15

3 Results for the linear regression model showing the relationship between calling somatic insertion/deletion mutations (SIM) and the QC measures. 16

4 Results for the linear regression model showing the relationship between calling somatic structural mutations (SStM) and the QC measures. 17

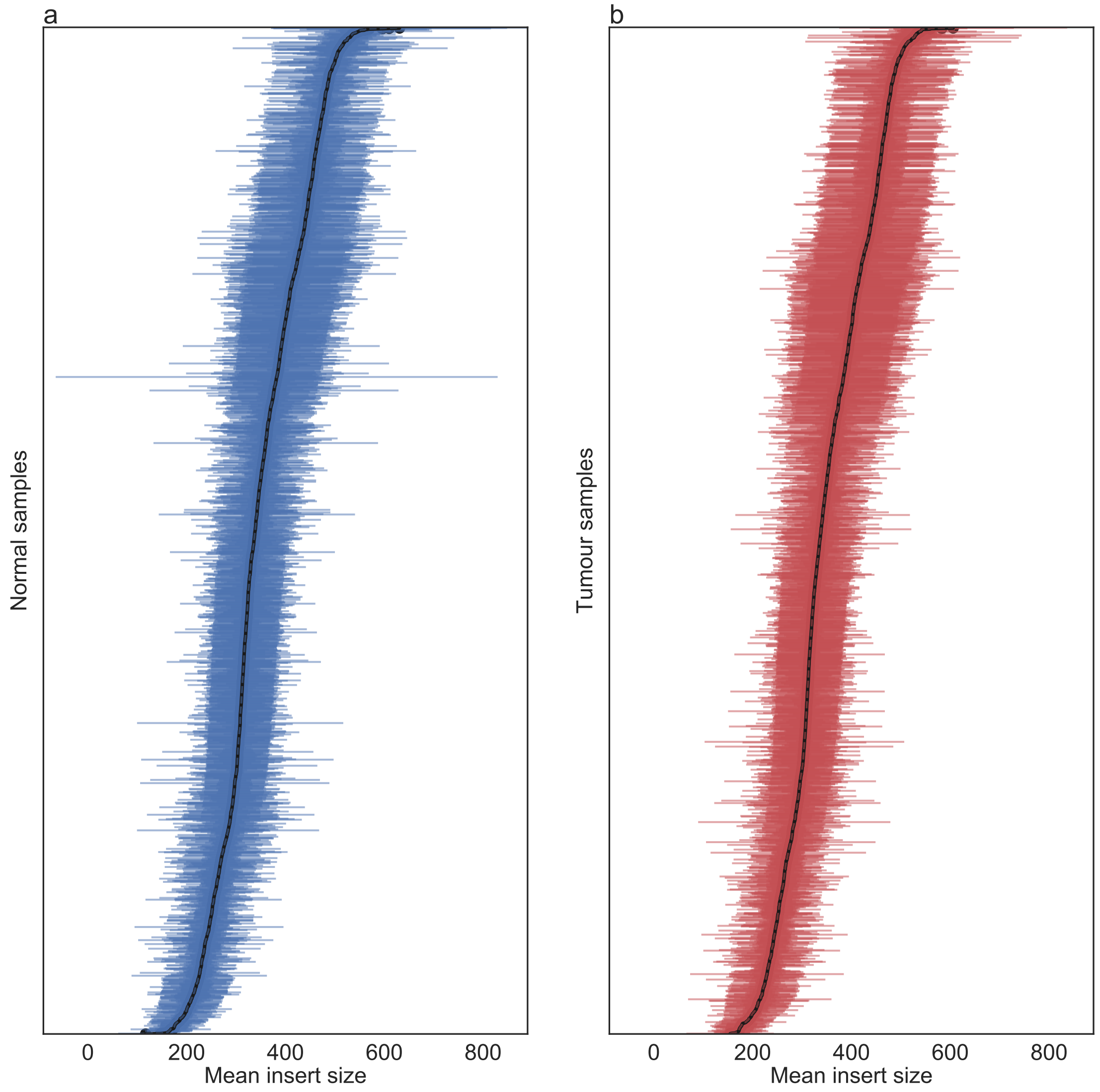

Figure 1: ***Mean insert size for (a) normal samples (n = 2,832) and (b) tumour samples (n = 2,959).*** *Each sample's mean insert size is represented by a black point, with an error bar, representing the standard deviation for each sample, centred on it. For the majority of the samples, given the large insert size and small read length (mostly around 100bp), it suggests there is very little overlap between the paired reads. The one outlier sample in (a) shown with standard deviation > 400bp, has over 50% of its paired reads mapping to different chromosomes, suggesting a different problem then overlapping reads.*

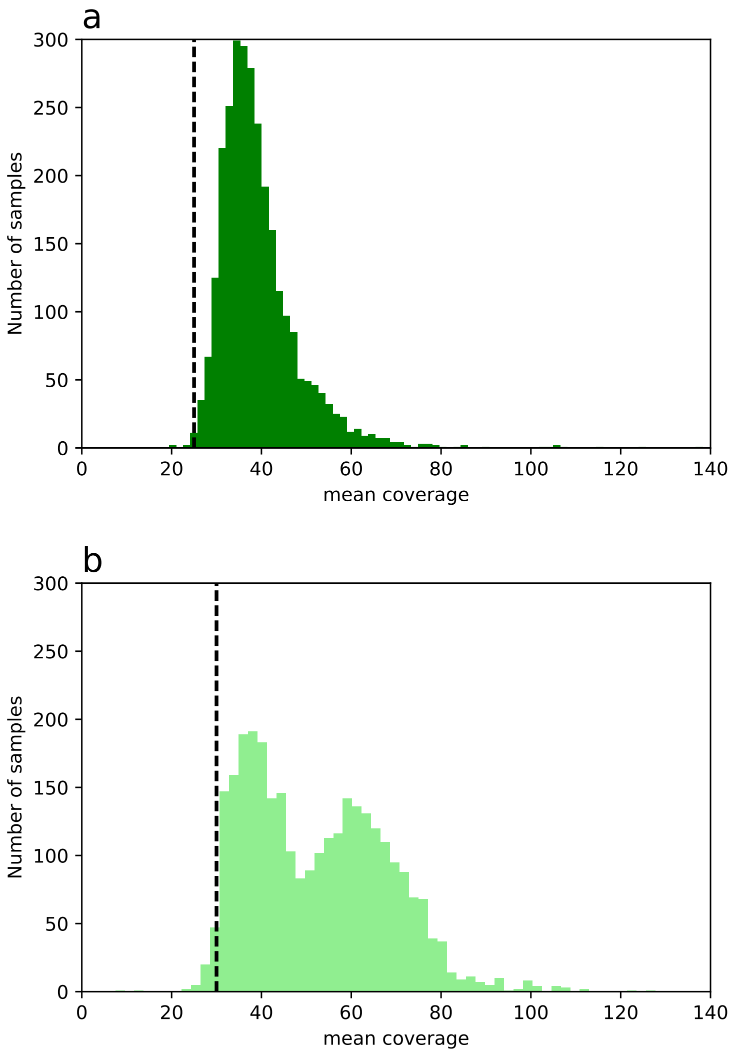

Figure 2: ***Histogram of the mean coverage for (a) normal samples (n = 2,832) and (b) tumour samples (n = 2,959).*** *Dashed lines represents the thresholds for normal (25×) and tumour (30×), above which the samples have reached the minimum mean coverage to pass this QC measure. The normal samples peak around 38× while the tumour samples have a bimodal distribution with maxima at 38× and 60×.*

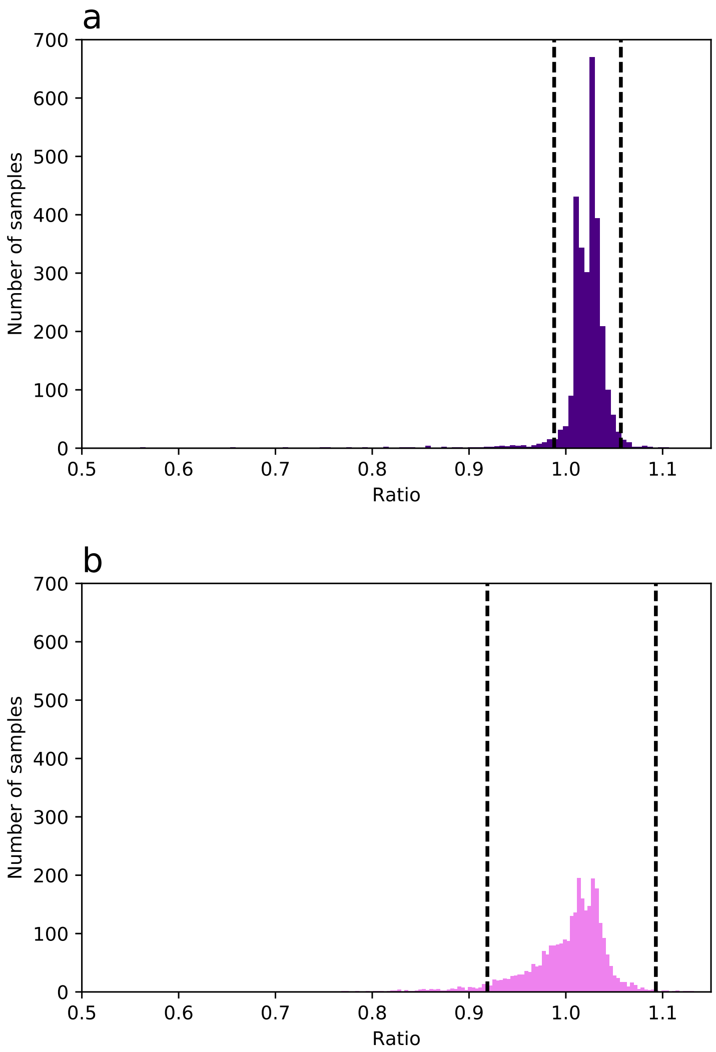

Figure 3: ***Histogram of the median over mean coverage ratios for (a) normal samples (n = 2,832) and (b) tumour samples (n = 2,959).*** *Dashed lines represent the thresholds for normal* $(0.99-1.06)$ *and tumour* $(0.92-1.09)$*, outside of which the samples are considered to have uneven coverage. Using the same scale for both graphs, the greater spread of the values for the tumour samples compared to the normal samples is apparent.*

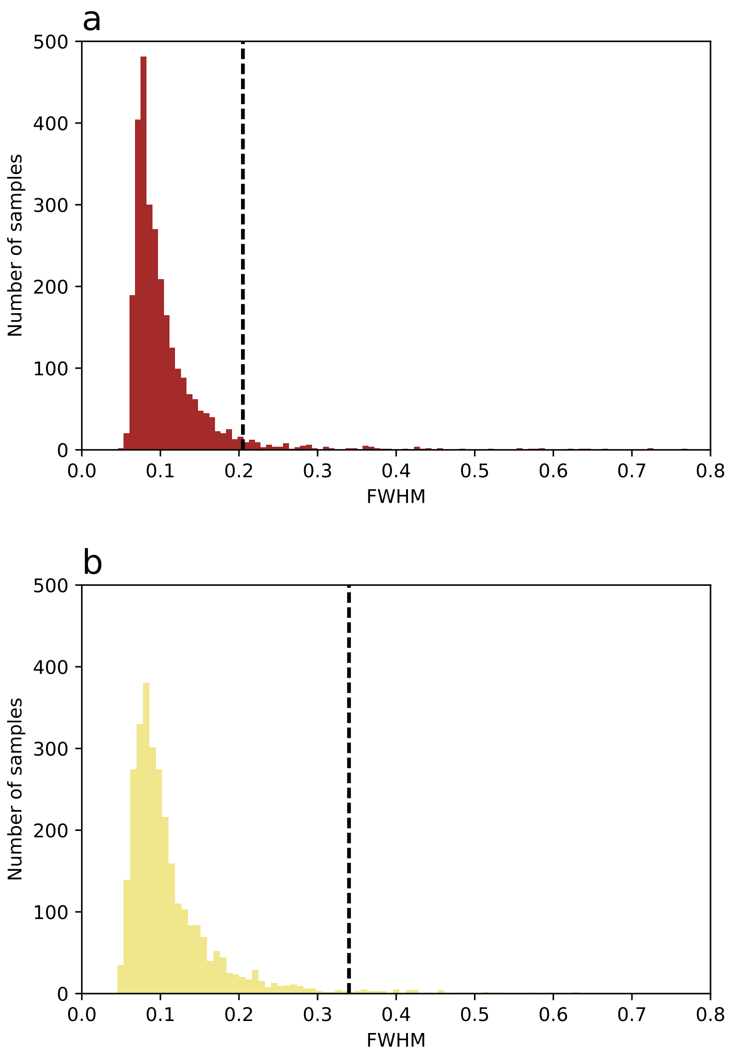

Figure 4: ***Histogram of the FWHM measures for (a) normal samples (n = 2,832) and (b) tumour samples (n = 2,959).*** *Dashed lines represents the thresholds for normal (0.20) and tumour (0.34), above which the samples are considered to have uneven coverage.*

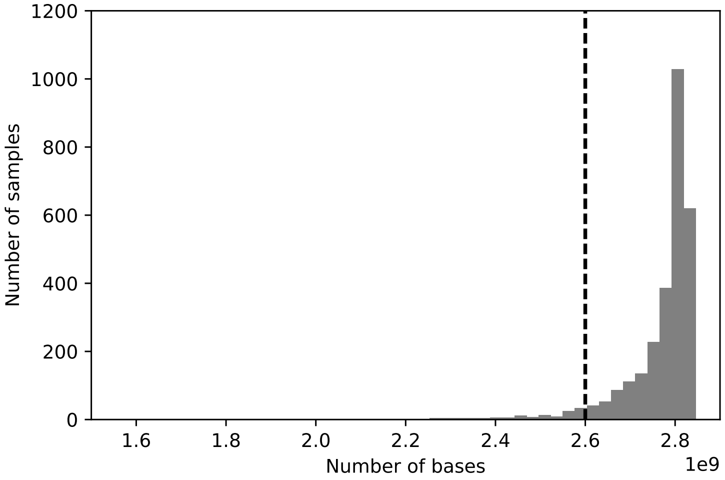

Figure 5: ***Histogram of the somatic mutation calling coverage values for normal-tumour sample pairs (n = 2,959).*** *The dashed line shows threshold (2.6×10^9 bases) for this measure, above which the normal-tumour sample is considered to have passed this QC measure.*

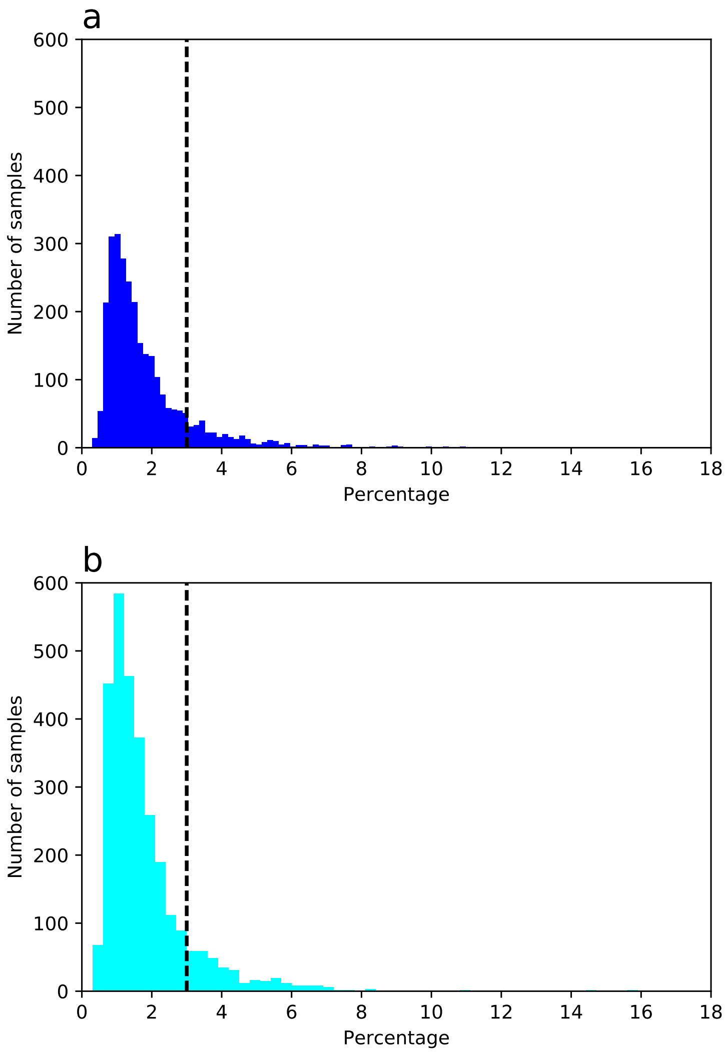

Figure 6: ***Histogram of the percentage of paired reads mapping to different chromosomes for (a) normal samples (n = 2,832) and (b) tumour samples (n = 2,959).*** *Dashed lines represents the threshold (3%) for both normal and tumour, below which the samples are considered to have passed this QC measure. Nine extreme outliers (7 in the normal and 2 in the tumour with values greater than 18%) are not shown.*

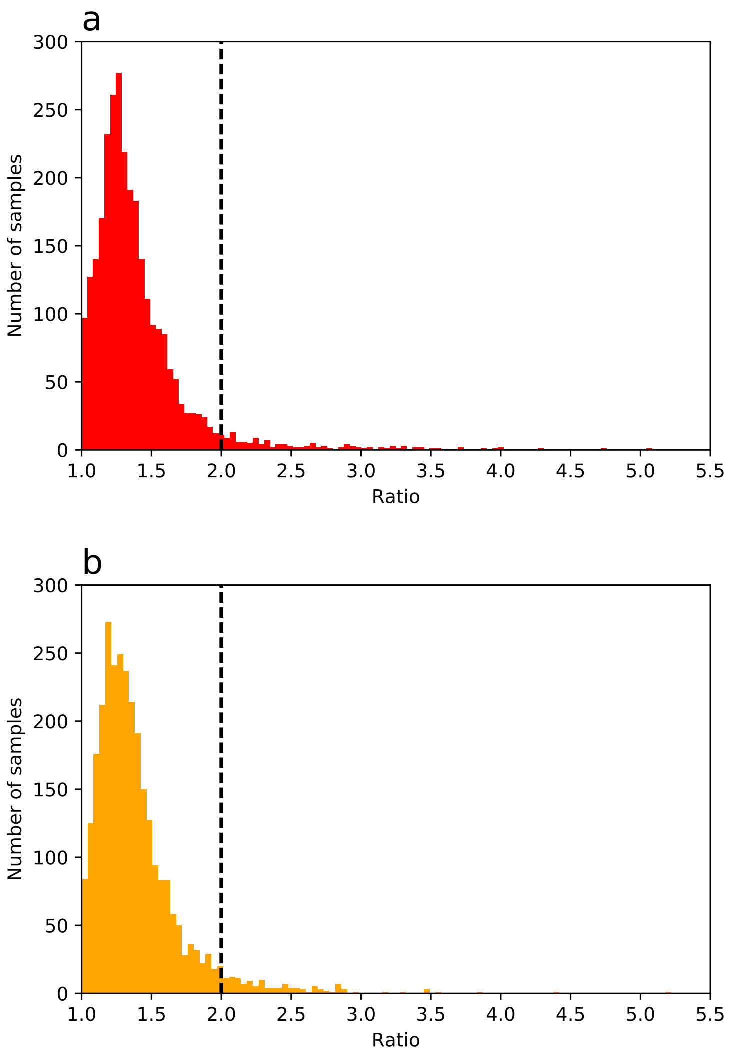

Figure 7: ***Histogram of the ratios of difference in edits between paired reads for (a) normal samples (n = 2,832) and (b) tumour samples (n = 2,959).*** *Dashed lines represents the threshold for normal and tumour, below which the samples are considered to pass this QC measure.*

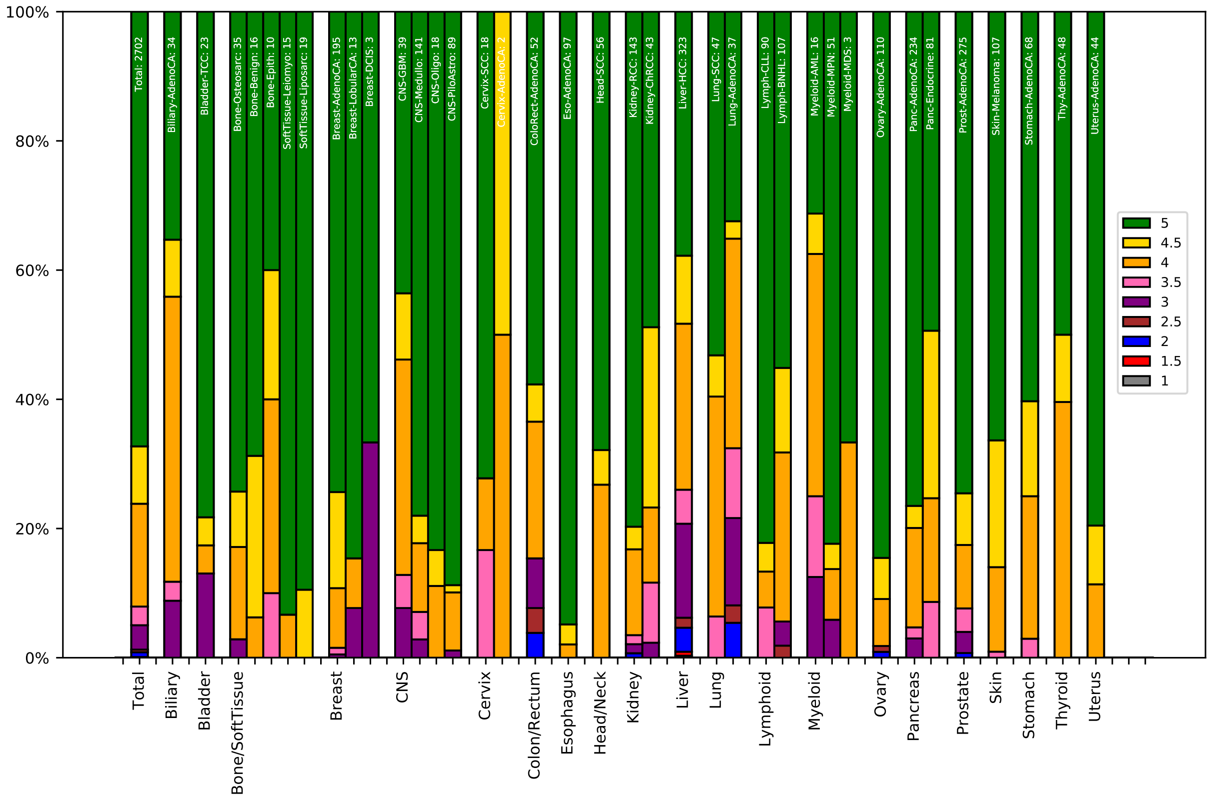

Figure 8: ***Histogram of the star ratings for the PCAWG genomes, as grouped by organ system (as labelled along the x-axis), and then the specific tumour type.*** *The tumour type and number of samples in the project are labelled at the top of the bar. The total of 2,702 tumour samples, does not include donors that are missing histological data. (as opposed to 2,959 tumour samples used in other graphs). This graphs suggests that the difference of quality was not due to sequencing of different organ systems or tumour types.*

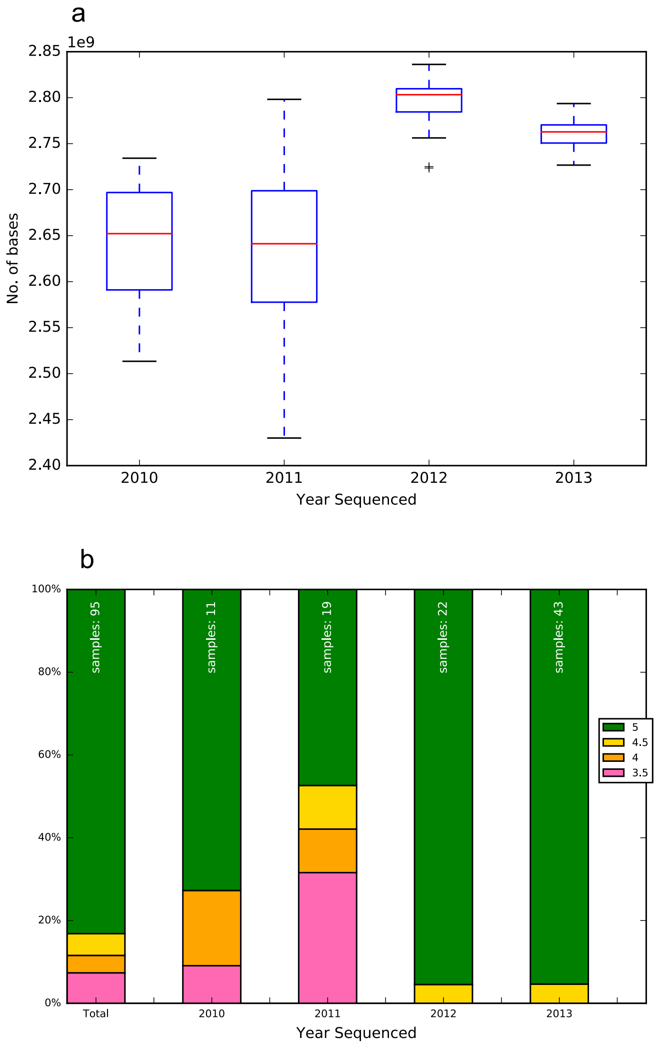

Figure 9: ***Increase in quality of the CLLE-ES samples sequenced in 2012/13 compared to 2010/11.*** *This increase in quality is shown in (a) the somatic mutation calling coverage and (b) the star rating (both with n = 95 samples). This can be explained by the change to a no-PCR sample preparation protocol. Note that these plots do not include results from 5 samples in the CLLE-ES project for which we do not have the associated detailed metadata. The box plots show the somatic mutation calling coverage for each sample, with median, the 25% and 75% quartiles shown by the box and the whiskers illustrating 1.5 × the interquartile range, with outliers highlighted outside this range.*

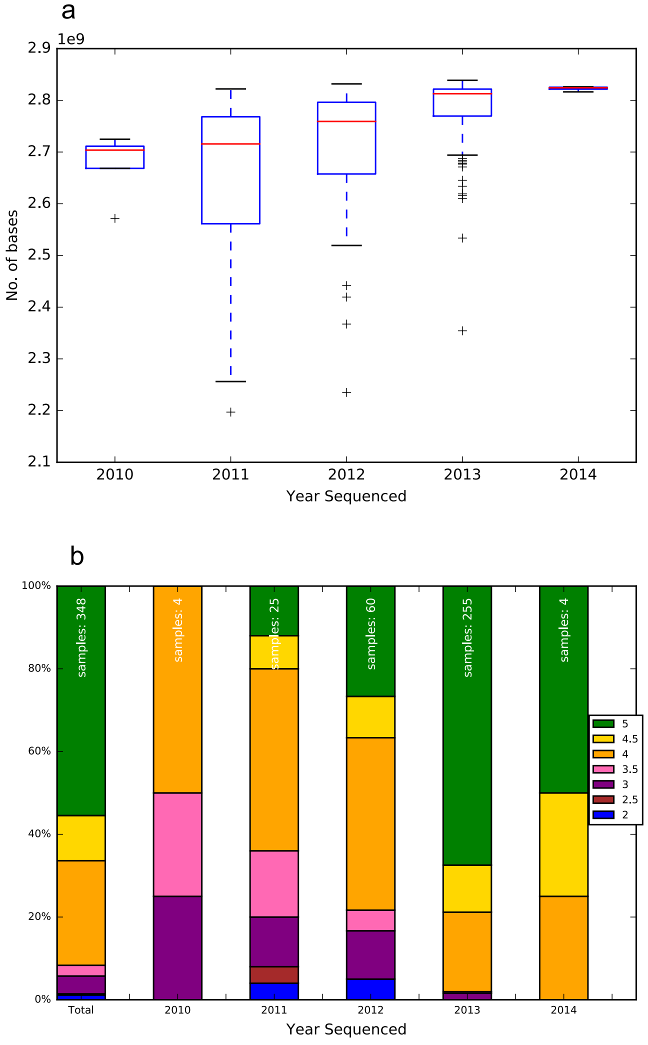

Figure 10: ***Quality of the samples sequenced in the Broad Institute over time.*** *As shown by (a) the somatic mutation calling coverage and (b) the star rating (both with n = 348 samples) there is a measurable increase in quality in both the number of positions with sufficient coverage to call a somatic mutation and the other all star count. Possible causes may be the instrument that was used (earlier sequencing was more likely done using Illumina GAII and later sequencing Illumina Hiseq) and/or improved experimental procedures. The box plots show the somatic mutation calling coverage for each sample, with median, the 25% and 75% quartiles shown by the box and the whiskers illustrating 1.5 $\times$ the interquartile range, with outliers highlighted outside this range.*

	p-value	corrected p-value	Mann-Whitney U statistic
SSM	1.31E-04	1.62E-04	319861
SIM	1.62E-04	1.62E-04	319273
SStM	3.87E-18	1.16E-17	384010

Table 1: ***Table showing that somatic mutation callers were in greater agreement for cancer genome sequences with 4 stars or more.*** *A two-sided Mann-Whitney-U test was used and corrected for multiple testing using the Benjamini/Hochberg method with FDR = 0.05. The results suggest that the higher the quality the samples (based on our methods) the more likely there is concordance between different somatic mutation callers for single base (SSM), insertion/deletion (SIM) and structural mutations (SStM).*

	Estimate	Std. Error	t value	Pr(>|t|)	adj pvalue
(Intercept)	8.99E-01	1.73E-01	5.19E+00	2.23E-07	1.34E-06
Mean normal	-3.77E-04	4.46E-04	-8.45E-01	3.98E-01	5.19E-01
Mean tumour	2.45E-03	3.02E-04	8.11E+00	7.63E-16	2.29E-14
FWHM normal	-2.13E-02	7.52E-02	-2.83E-01	7.77E-01	8.04E-01
FWHM tumour	-2.11E-01	6.42E-02	-3.28E+00	1.06E-03	2.89E-03
CallPow	-1.15E-10	6.61E-11	-1.74E+00	8.24E-02	1.37E-01
DiffChrom normal	6.31E-03	2.80E-03	2.25E+00	2.44E-02	4.58E-02
DiffChrom tumour	-1.43E-02	2.68E-03	-5.34E+00	1.03E-07	1.03E-06
RatioEdits normal	1.36E-02	1.24E-02	1.10E+00	2.72E-01	3.70E-01
RatioEdits tumour	-8.20E-03	1.39E-02	-5.89E-01	5.56E-01	6.67E-01

Table 2: ***Results for the linear regression model showing the relationship between calling somatic single base mutations (SSM) and the QC measures.*** *Shown is the coefficient estimate, the standard error, the t-statistic, p-values for a two sided t-test and the adjusted p-value (using the Benjamini/Hochberg method for all p-values reported in Supplementary Tables 2-4 with FDR = 0.05). The multiple R-squared value is 0.0473 and adjusted R-squared 0.0441, suggesting this linear regression model only explains a small amount of the variance in the data.*

	Estimate	Std. Error	t value	Pr(>|t|)	adj pvalue
(Intercept)	5.52E-01	1.13E-01	4.89E+00	1.08E-06	5.40E-06
Mean normal	3.28E-04	2.91E-04	1.13E+00	2.60E-01	3.70E-01
Mean tumour	4.60E-04	1.97E-04	2.34E+00	1.94E-02	3.87E-02
FWHM normal	-3.68E-02	4.91E-02	-7.50E-01	4.54E-01	5.67E-01
FWHM tumour	-1.45E-01	4.19E-02	-3.46E+00	5.56E-04	1.67E-03
CallPow	-8.38E-11	4.31E-11	-1.94E+00	5.20E-02	9.18E-02
DiffChrom normal	-9.20E-05	1.83E-03	-5.00E-02	9.60E-01	9.60E-01
DiffChrom tumour	-6.05E-03	1.74E-03	-3.47E+00	5.24E-04	1.67E-03
RatioEdits normal	2.69E-03	8.06E-03	3.34E-01	7.38E-01	8.04E-01
RatioEdits tumour	-2.52E-02	9.09E-03	-2.77E+00	5.67E-03	1.31E-02

Table 3: ***Results for the linear regression model showing the relationship between calling somatic insertion/deletion mutations (SIM) and the QC measures.*** *Shown is the coefficient estimate, the standard error, the t-statistic, p-values for a two sided t-test and the adjusted p-value (using the Benjamini/Hochberg method for all p-values reported in Supplementary Tables 2-4 with FDR = 0.05). The multiple R-squared value is 0.0161 and adjusted R-squared 0.0123, suggesting this linear regression model only explains a small amount of the variance in the data.*

	Estimate	Std. Error	t value	Pr(>|t|)	adj pvalue
(Intercept)	5.43E-01	2.04E-01	2.67E+00	7.68E-03	1.65E-02
Mean normal	-1.69E-03	5.24E-04	-3.23E+00	1.24E-03	3.10E-03
Mean tumour	-5.66E-04	3.67E-04	-1.54E+00	1.23E-01	1.95E-01
FWHM normal	1.11E-01	8.80E-02	1.26E+00	2.07E-01	3.10E-01
FWHM tumour	-3.97E-01	7.59E-02	-5.23E+00	1.86E-07	1.34E-06
CallPow	2.43E-11	7.80E-11	3.12E-01	7.55E-01	8.04E-01
DiffChrom normal	-1.47E-02	3.33E-03	-4.41E+00	1.09E-05	4.67E-05
DiffChrom tumour	-2.62E-02	3.73E-03	-7.04E+00	2.55E-12	3.82E-11
RatioEdits normal	5.64E-03	1.51E-02	3.75E-01	7.08E-01	8.04E-01
RatioEdits tumour	-6.61E-02	1.65E-02	-4.01E+00	6.33E-05	2.37E-04

Table 4: ***Results for the linear regression model showing the relationship between calling somatic structural mutations (SStM) and the QC measures.*** *Shown is the coefficient estimate, the standard error, the t-statistic, p-values for a two sided t-test and the adjusted p-value (using the Benjamini/Hochberg method for all p-values reported in Supplementary Tables 2-4 with FDR = 0.05). The multiple R-squared value is 0.0787 and adjusted R-squared 0.0754, suggesting this linear regression model only explains a small amount of the variance in the data.*
