# Supplementary material for: Framework for quality assessment of whole genome cancer sequences
Source: Nat Commun. 2020 Oct 7;11:5040. doi: 10.1038/s41467-020-18688-y (PMC7541455; doi:10.1038/s41467-020-18688-y)
Supplement: Supplementary file 3 — Reporting Summary [file 41467_2020_18688_MOESM3_ESM.pdf]

nature research

Corresponding author(s): Dr Ivo G. Gut

Last updated by author(s): Mar 2, 2020

Reporting Summary

Nature Research wishes to improve the reproducibility of the work that we publish. This form provides structure for consistency and transparency in reporting. For further information on Nature Research policies, see Authors & Referees and the Editorial Policy Checklist.

Statistics

For all statistical analyses, confirm that the following items are present in the figure legend, table legend, main text, or Methods section.

n/a	Confirmed	
☐	☒	The exact sample size (*n*) for each experimental group/condition, given as a discrete number and unit of measurement
☐	☒	A statement on whether measurements were taken from distinct samples or whether the same sample was measured repeatedly
☐	☒	The statistical test(s) used AND whether they are one- or two-sided<br>*Only common tests should be described solely by name; describe more complex techniques in the Methods section.*
☐	☒	A description of all covariates tested
☐	☒	A description of any assumptions or corrections, such as tests of normality and adjustment for multiple comparisons
☐	☒	A full description of the statistical parameters including central tendency (e.g. means) or other basic estimates (e.g. regression coefficient) AND variation (e.g. standard deviation) or associated estimates of uncertainty (e.g. confidence intervals)
☐	☒	For null hypothesis testing, the test statistic (e.g. *F*, *t*, *r*) with confidence intervals, effect sizes, degrees of freedom and *P* value noted<br>*Give P values as exact values whenever suitable.*
☒	☐	For Bayesian analysis, information on the choice of priors and Markov chain Monte Carlo settings
☒	☐	For hierarchical and complex designs, identification of the appropriate level for tests and full reporting of outcomes
☐	☒	Estimates of effect sizes (e.g. Cohen's *d*, Pearson's *r*), indicating how they were calculated

Our web collection on statistics for biologists contains articles on many of the points above.

Software and code

Policy information about availability of computer code

Data collection	The data was not downloaded. The analysis was done in the servers containing the PCAWG data.
Data analysis	To calculate the quality control measures, we have provided a a Docker Container, which can be found at https://dockstore.org/containers/quay.io/jwerner_dkfz/pancanqc:1.2.2 PanCanQC . To calculate the star rating and illustrate the results graphically, we have provided python notebooks at github.com/jpwhalley/PCAWG-QC_Graphs .

For manuscripts utilizing custom algorithms or software that are central to the research but not yet described in published literature, software must be made available to editors/reviewers. We strongly encourage code deposition in a community repository (e.g. GitHub). See the Nature Research guidelines for submitting code & software for further information.

Data

Policy information about availability of data

All manuscripts must include a data availability statement. This statement should provide the following information, where applicable:

- Accession codes, unique identifiers, or web links for publicly available datasets
- A list of figures that have associated raw data
- A description of any restrictions on data availability

The whole genome sequences used in this paper can be downloading from the PCAWG page in the ICGC Data Portal: dcc.icgc.org/pcawg . No accession codes are needed, as only data from the PCAWG project is provided on this page in the portal.

Field-specific reporting

Please select the one below that is the best fit for your research. If you are not sure, read the appropriate sections before making your selection.

☒ Life sciences ☐ Behavioural & social sciences ☐ Ecological, evolutionary & environmental sciences

For a reference copy of the document with all sections, see nature.com/documents/nr-reporting-summary-flat.pdf

Life sciences study design

All studies must disclose on these points even when the disclosure is negative.

Sample size	We worked with the Pan-Cancer Analysis of Whole Genomes (PCAWG), which at the time was the largest collection of whole cancer genomes at the time (2,959 normal-tumour genome pairs). The cohort looked to collect all available ICGC or TCGA whole cancer genomes sequenced using using Illumina Genome Analyzer II or HiSeq by 2014. No statistical tests were done to predetermine sample size.
Data exclusions	Due to our quality control measures, samples were later excluded from the overall PCAWG. However we have kept them in this study to show the range of quality the whole cancer genomes.
Replication	Our quality measures were defined for whole, genome sequences for 48 individual projects from 18 sequencing centres. No project or sequencing centre provided data in which the quality measures could not be defined or used in downstream analyses.
Randomization	This was not relevant for our work as we did not have a case/control set up. We were looking to define the quality of the data, to reassure the researchers working with it, that it was valid to be used in their analyses.
Blinding	As explained above in Randomization, we did not allocate a our data to separate groups.

Reporting for specific materials, systems and methods

We require information from authors about some types of materials, experimental systems and methods used in many studies. Here, indicate whether each material, system or method listed is relevant to your study. If you are not sure if a list item applies to your research, read the appropriate section before selecting a response.

Materials & experimental systems

n/a	Involved in the study	
☒	☐	Antibodies
☒	☐	Eukaryotic cell lines
☒	☐	Palaeontology
☒	☐	Animals and other organisms
☐	☒	Human research participants
☒	☐	Clinical data

Methods

n/a	Involved in the study	
☒	☐	ChIP-seq
☒	☐	Flow cytometry
☒	☐	MRI-based neuroimaging

Human research participants

Policy information about studies involving human research participants

Population characteristics	This data is from the Pan-Cancer Analysis of Whole Genomes (PCAWG), of which the marker paper - https://rdcu.be/b2iQo - describes the demographics and ancestry of the population. To summaries of the cohort consisted of 55% males and 45% females with a mean age of 56 years (range, 1-90 years). Based on the germline sequencing the the biggest two populations in the cohort are those of European descent (77%) and those of East Asians descent (16%). The dataset contains 38 distinct tumour types.
Recruitment	Patients were recruited by local centres following local protocol.
Ethics oversight	The consortium was overseen by both the TCGA and ICGC.

Note that full information on the approval of the study protocol must also be provided in the manuscript.
