# Supplementary material for: Framework for quality assessment of whole genome cancer sequences
Source: Nat Commun. 2020 Oct 7;11:5040. doi: 10.1038/s41467-020-18688-y (PMC7541455; doi:10.1038/s41467-020-18688-y)
Supplement: Supplementary file 4 — Description of Additional Supplementary Files [file 41467_2020_18688_MOESM4_ESM.pdf]

Description of Additional Supplementary Files

File Name: Supplementary Data 1
Description: Quality Control measures' values and star rating for all 2,959 whole genome, cancer samples tested.

File Name: Supplementary Data 2
Description: Dataset linking the 48 individual projects, that make up this cohort, to their tumour type.
